# Case of Malformation of the Heart, in Which Death Resulted from Obstruction of the Trunk of the Pulmonary Artery

**Published:** 1847-09

**Authors:** Thomas B. Peacock


*Case of Malformation of the Heart, in which death resulted from Obstruction of the Trunk of the Pulmonary Artery.* By THOMAS B. PEACOCK, M. D. The subject of this case was a boy, fifteen years of age, who had from early life exhibited slight appearances of cyanosis. Twelve months before his death he was thrown from a cart, and after that period the lividity of the countenance became much more marked, and he was subject to frequent attacks of chest affection, and to palpitation. His last illness was of eight days' duration, and commenced with rheumatic symptoms, followed by difficulty of breathing and pain in the chest. When admitted into the Royal Free Hospital, he exhibited very marked cyanosis, and a loud murmur, accompanying the impulse of the heart, was audible in the præcordia, and along the sternum. At the situation of the base of the heart the murmur masked all other sounds; but toward the apex, and the lower and right side of the sternum, it was followed by a loud second sound. He died a few hours after his admission. On examination, the aorta was found to arise in part from the right ventricle, and the orifice and trunk of the pulmonary artery were of very small size. The right ventricle was also separated by a supernumerary septum into two cavities, one being the infundibular portion, giving origin, as usual, to the pulmonary artery; the other consisting of the sinus of the ventricle, communicating with the aorta. The abnormal septum was incomplete over a space about equal in size to that by which the aorta communicated with the right ventricle. The orifice of the pulmonary artery was provided with only two valves, and these were so thick and rigid as to occasion a further contraction of the aperture. The trunk of the vessel was entirely obstructed by partially decolorized coagula, which were laminated and adherent to the thickened and diseased valves and coats of the vessels. The author remarked that the case was closely allied to the ordinary form of malformation in which, with congenital contraction of the pulmonary artery, the septum of the ventricles is defective at the base, so as to allow the aorta to receive its supply of blood from both ventricles. Its great interest, however, lay in the division of the right ventricle into two cavities communicating with the aorta and pulmonary artery respectively, and the free admixture of the venous and arterial currents of blood, which must consequently have taken place throughout the whole of the boy's life. So far, however, from this having been productive of marked cyanosis, it was stated that there was but little peculiarity in the boy's appearance till the supervention of secondary disease in the pulmonary artery after the fall, twelve months before his death. The cause of death was also, it was remarked, unusual. Though obstructions in the terminal branches of the pulmonary artery had been shown by Baron and Paget to be of frequent occurrence, the writer was not aware of any other case in which the trunk of that vessel had become obstructed.—*Lancet.*

